# Circadian Changes of Dendritic Spine Geometry in Mouse Barrel Cortex

**DOI:** 10.3389/fnins.2020.578881

**Published:** 2020-09-29

**Authors:** Malgorzata Jasinska, Olga Woznicka, Ewa Jasek-Gajda, Grzegorz J. Lis, Elzbieta Pyza, Jan A. Litwin

**Affiliations:** ^1^Department of Histology, Jagiellonian University Medical College, Krakow, Poland; ^2^Department of Cell Biology and Imaging, Institute of Zoology and Biomedical Research, Jagiellonian University, Krakow, Poland

**Keywords:** circadian rhythmicity, influence of light, dendritic spine morphology, neural plasticity, electron microscopy, somatosensory cortex

## Abstract

The circadian rhythmicity changes the density and shape of dendritic spines in mouse somatosensory barrel cortex, influencing their stability and maturation. In this study, we analyzed the main geometric parameters of dendritic spines reflecting the strength of synapses located on these spines under light/dark (12:12) and constant darkness conditions, in order to distinguish between endogenously regulated and light-driven parameters. Using morphological analysis of serial electron micrographs, as well as three-dimensional reconstructions, we found that the light induces elongation of single-synapse spine necks and increases in the diameter of double-synapse spine necks, increasing and decreasing the isolation of synapses from the parent dendrite, respectively. During the subjective night of constant darkness, we observed an enlargement of postsynaptic density area in inhibitory synapses and an increase in the number of polyribosomes inside double-synapse spines. The results show that both endogenous effect (circadian clock/locomotor activity) and light affect the morphological parameters of single- and double-synapse spines in the somatosensory cortex: light reduces the efficiency of excitatory synapses on single-synapse spines, increases the effect of synaptic transmission in double-synapse spines, and additionally masks the endogenous clock-driven enlargement of inhibitory synapses located on double-synapse spines. This indicates a special role of double-synapse spines and their inhibitory synapses in the regulation of synaptic transmission during both circadian and diurnal cycles in the mouse somatosensory cortex.

## Introduction

The quantitative and qualitative changes of small dendritic protrusions—dendritic spines—associated with activity-dependent neural plasticity have been observed in various areas of the brain. The importance of such changes results from the general belief that increase/decrease in the number and size of dendritic spines is associated with memory formation processes (Kasai et al., 2010). The morphological modifications of spines could be even more significant than their numerical changes (Geinisman et al., 2000, 2001), because they provide an observable reflection of spine function (Yuste et al., 2000). The shape of dendritic spines is highly flexible and can be modified in a relatively short time by experience-dependent synaptic plasticity: long-term depression, long-term potentiation (Nikonenko et al., 2002; Bourne and Harris, 2008; Bosch and Hayashi, 2012), or associative learning process (Leuner and Shors, 2004; Bourne and Harris, 2008; Kasai et al., 2010; Jasinska et al., 2016).

The density and morphology of spines are additionally dependent on the activity of animal during the 24-h period (sleep/wakefulness) (Maret et al., 2011; Yang and Gan, 2012; Acosta-Peña et al., 2015; Havekes et al., 2016) or on the phase of the circadian cycle (day/night) (Ikeno et al., 2013; Liston et al., 2013; Jasinska et al., 2015, 2019). While studies of the geometric parameters of the spines during sleep/wake cycle are more frequent (Frank and Cantera, 2014; van der Zee, 2015; Areal et al., 2017; Raven et al., 2018), the information on morphological changes of the spines in the circadian rhythm is relatively scarce. Investigation of such changes seems to be important, because circadian rhythmicity affects synaptic plasticity irrespective of sleep/wake state (Frank and Cantera, 2014).

Circadian neural plasticity has been extensively studied in insects and focused on the visual system, as well as the motor neurons (Frank, 2016; Krzeptowski et al., 2018). The diurnal and circadian changes were found in various synapses (Pyza and Meinertzhagen, 1993; Górska-Andrzejak et al., 2013; Ruiz et al., 2013; Woźnicka et al., 2015), as well as in the size and morphology of axons, dendrites, or even whole neurons (Pyza and Meinertzhagen, 1999; Górska-Andrzejak et al., 2005; Mehnert et al., 2007; Fernández et al., 2008).

In rodents, cyclic morphological modifications of synapses and neurons were observed in the superchiasmatic nucleus—a structure directly responsible for synchronization of the circadian rhythmicity in the body (Becquet et al., 2008; Girardet et al., 2010), as well as in the retina (Behrens et al., 1998; Balkema et al., 2001). The studies of dendritic spines related to diurnal or circadian plasticity are limited to the hippocampus and some areas of the neocortex. It has been shown that under light/dark (LD) conditions dendritic spines are more numerous during the active phase of animals in rat hippocampus (Ikeda et al., 2015) and infralimbic cortex (Perez-Cruz et al., 2009b), as well as in mouse motor cortex (Liston et al., 2013). It seems that circadian oscillations of glucocorticoids affect the dynamics of spines in the motor and somatosensory cortex (Liston and Gan, 2011; Liston et al., 2013). The peak of glucocorticoid release occurs at the beginning of the active phase of animals (Cheifetz, 1971; Chung et al., 2011), when intensified dendritic spine formation is also observed (Liston and Gan, 2011).

In the mouse barrel cortex, the density of spines with single excitatory synapses (single-synapse spines) increases in the day under LD conditions, whereas the number of double-synapse spines is higher during the night/subjective night (LD/constant darkness, DD), what indicates light-dependent regulation of single-synapse spine density and endogenous regulation of the number of double-synapse spines (Jasinska et al., 2015). The increase in the density of single- and double-synapse spines is accompanied by a simultaneous increase in the number of excitatory and inhibitory synapses, respectively (Jasinska et al., 2014; Jasinska et al., 2015).

Diurnal changes in the density of dendritic spines appear to be closely related to the photoperiod (long and short day) and brain subregion (CA1/DG hippocampus; Ikeno et al., 2013) and can even fluctuate between the hemispheres of the brain (left/right; Perez-Cruz et al., 2009a).

In the mouse barrel cortex, the shape modifications of single-synapse spines are driven by the circadian clock, whereas in double-synapse spines they remain under the influence of the light (Jasinska et al., 2019).

The light also promotes enlargement and maturation of both single- and double-synapse spines. In the rest phase, an increase in the number of mushroom single-synapse spines was observed under LD conditions in mouse somatosensory cortex, whereas double-synapse spines contained spine apparatus (SA) indicating their maturity (Jasinska et al., 2019). In hippocampal CA1 field, the number of spines with large heads increased at the beginning of the active phase (Ikeda et al., 2015).

Dendritic spine is usually described as a structure with a distinct head and neck, although the degree of head/neck distinction is variable and depends on the overall shape of the spine (Peters and Kaiserman-Abramof, 1970; Bourne and Harris, 2007). It is widely believed that the head is functionally more important than the neck, probably due to the fact that the majority of excitatory synapses are located on the spine heads (Maiti et al., 2015). This rule, however, is not fully justified in case of spines with two different synapses (double-synapse spines), located in mouse somatosensory cortex, in which the excitatory synapses are usually located on the spine heads, while the inhibitory synapses on the spine necks (Jasinska et al., 2006).

Dendritic spines are separate biochemical compartments allowing control of protein flow, compartmentalization of calcium (Denk et al., 1995; Kovalchuk et al., 2000; Noguchi et al., 2005), and regulation of second messenger diffusion between the head and the parent dendrite (Tønnesen and Nägerl, 2016). The general morphology of a spine in which wide head is connected to the dendrite via narrow neck allows complete isolation of the spine from dendrite shaft or at least slows down diffusion between spine head and the shaft (Sorra and Harris, 2000; Bloodgood and Sabatini, 2005). Because of the barrier created by the spine neck, the concentration of calcium in the head can reach a higher level than in the parent dendrite (Korkotian and Segal, 2007; Segal, 2010), what might be an effective way of protecting the neuron from overstimulation or excitotoxicity (Sorra and Harris, 2000; Segal, 2010).

The spine necks can also electrically separate the spines (Araya et al., 2014) by isolating the inputs of synapses located on adjacent spines, as well as isolating them from dendrite (Araya et al., 2006). It suggests that spine neck might play a significant role in the regulation of synapse effectiveness.

Length and diameter of spine neck are important for neck resistance (Wickens, 1988; Spacek and Harris, 1997). Interestingly, no correlation has been found between these parameters, and it seems that they are regulated independently (Arellano et al., 2007). The increase in the spine neck length reduces the strength of the synapse (Araya et al., 2014). It has been shown that excitatory postsynaptic potential amplitudes are inversely proportional to spine neck length, as well as that spines with long necks have a weak or negligible contribution to somatic voltage, but after synaptic stimulation, they could shorten the necks and increase synaptic efficiency (Araya et al., 2014). Parameters of the spine neck (Fifkova and Anderson, 1981; Jasinska et al., 2006; Tønnesen et al., 2014).

The earlier studies of the circadian rhythm of spine morphology were focused on spine shapes, which indicate the level of spine stability, but do not provide information about the geometric parameters of the spines, which indirectly influence the strength and efficiency of spine-associated synapses. In our previous study, we analyzed the influence of the circadian clock/locomotor activity and light on the shape of spines (stubby, thin, and mushroom) and their content [smooth endoplasmic reticulum (sER), SA] reflecting the level of spine maturity and stability. In the present exploratory study, we investigate circadian changes of measurable geometric parameters (spine length and volume, spine head diameter, spine neck length, and diameter), as well as postsynaptic density (PSD) area indicative of synapse strength and the number of polyribosomes reflecting the local protein synthesis, which plays a key role in the modification of synapses located on the spines.

## Materials and Methods

We examined the same collection of ultrathin sections, which was used in our previous study (Jasinska et al., 2019).

### Animals

The experiments were performed on C57BL male mice aged 5 to 6 weeks. This study was carried out in accordance with the Council Directive 2010/63EU of the European Parliament and the Council of 22 September 2010 on the protection of animals used for scientific purposes and approved by the Animal Care and Use Committees of the Jagiellonian University.

### Analysis of Locomotor Activity

All animal were kept for 2 weeks under the following conditions: LD 12:12 (12 h of light and 12 h of dark), light 60 lx, 25°C, and 50% humidity to get used to standard light conditions. Next, the mice were divided into LD (*n* = 8) and DD (*n* = 8) groups. The mice in the LD group were kept for the next 2 weeks under LD 12:12 and in the DD group—under constant darkness. The animals were fed a standard diet and water *ad libitum.*

From the beginning of experiments, mice stayed in the cages with free access to a running wheel coupled with a 16-channel electromagnetic pulse counter (MIKI 1; Autel, Poland). The running activity was continuously recorded: the number of wheel rotations per minute was counted and then transferred to PC computer by RS232 interface. Data were recorded and saved on a computer disk by using RealTerm software (RealTerm: Serial/TCP Terminal 2.0.0.70^1^). The obtained data were analyzed using NIH ImageJ ActogramJ software^2^.

All mice showing locomotor rhythmicity under LD conditions were selected for further experiments. Eight mice were killed 2 h after the beginning of the light phase or the subjective day (*n* = 4 per subgroup LD REST and DD REST), and eight mice were killed 2 h after the beginning of the dark phase or the subjective night (*n* = 4 per subgroups LD ACTIVE and DD ACTIVE).

### Transmission Electron Microscopy

The mice were anesthetized with Morbital (25–30 mg/kg of body weight; Biowet, Pulawy, Poland) and perfused through the heart with 20 mL of rinse buffer (0.2% glutaraldehyde and 2% paraformaldehyde in 0.1 M phosphate buffer, pH 7.4), followed by 100 to 150 mL of fixative (2.5% glutaraldehyde and 2% paraformaldehyde in 0.1 M phosphate buffer, pH 7.4). Immediately after perfusion, the brains were removed and kept in the same fixative for 24 h at 4°C.

Next, after a wash in 0.1 M phosphate buffer (pH 7.4), 60 μm tangential vibratome sections were cut from the barrel cortex region and examined under a stereomicroscope (Nikon Optiphot, Japan). Only sections containing the barrel field cortex were collected for further processing. The sections were washed in 0.1 M cacodylate buffer (pH 7.4), postfixed twice with 1% osmium tetroxide in 0.1 M cacodylate buffer, pH 7.4 (the first change containing 1.5% potassium ferrocyanide), washed in 70% ethanol containing 1% uranyl acetate, and after dehydration in graded series of ethanol, embedded in Epon (Polysciences, United States) between two silicon-coated glass slides.

The region of B2 barrel was identified according to the procedure described previously (Jasinska et al., 2010). The embedded slices were trimmed into blocks, and a series of 10 to 15 successive ultrathin sections (65–75 nm thick) were cut from each sample. The sections were collected on formvar-coated copper–palladium slot grids and contrasted with 1% lead citrate.

For examination of dendritic spine morphology, three to five series of electron micrographs (10–15 serial micrographs each) of the B2 barrel central area in which cell bodies are sparse were taken from each mouse at 30K magnification in JEOL JEM 2100 transmission electron microscope (JEOL, Japan). The micrographs were aligned using Adobe Photoshop CS software, and stacks of serial images were prepared.

### Morphological Analysis of Single- and Double-Synapse Spines

Only spines that were completely contained within each volume sample were selected for the quantitative analysis. Images of 201 single-synapse spines and 89 double-synapse spines from both LD and DD groups were chosen for morphological measurements and three-dimensional (3D) reconstruction (single synapse-spines—LD REST: 42, LD ACTIVE: 37, DD REST: 51, DD ACTIVE: 71; double synapse-spines—LD REST: 18, LD ACTIVE: 25, DD REST: 20, DD ACTIVE: 26).

**FIGURE 1:**
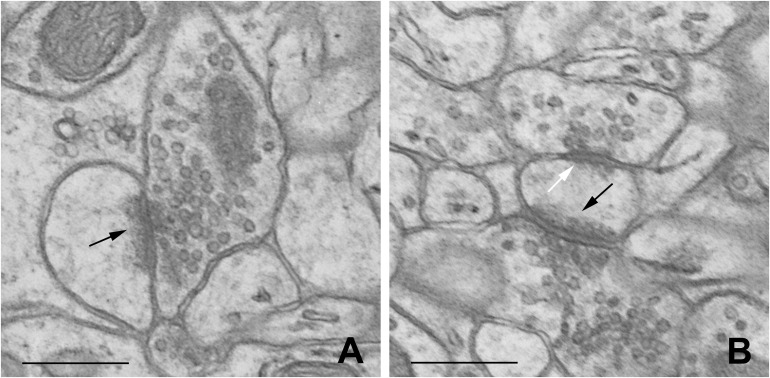
Representative electron micrographs of B2 barrel hollow showing single-synapse spine **(A)** and double-synapse spine **(B)**. Excitatory and inhibitory synapses are indicated by black and white arrows, respectively. Scale bars: 0.5 μm.

Dendritic spines were defined according to Knott et al. (2002). Synapses were characterized according to Jasinska et al. (2010, 2015). The distinction between excitatory and inhibitory synapses was based on the symmetry of synaptic membranes and on the appearance of synaptic vesicles (Figure 1).

3D reconstructions of the spines were performed using 3D Studio Max software (Discreet Logic, Montreal, QC, Canada) (Jasinska et al., 2016).

**FIGURE 2:**
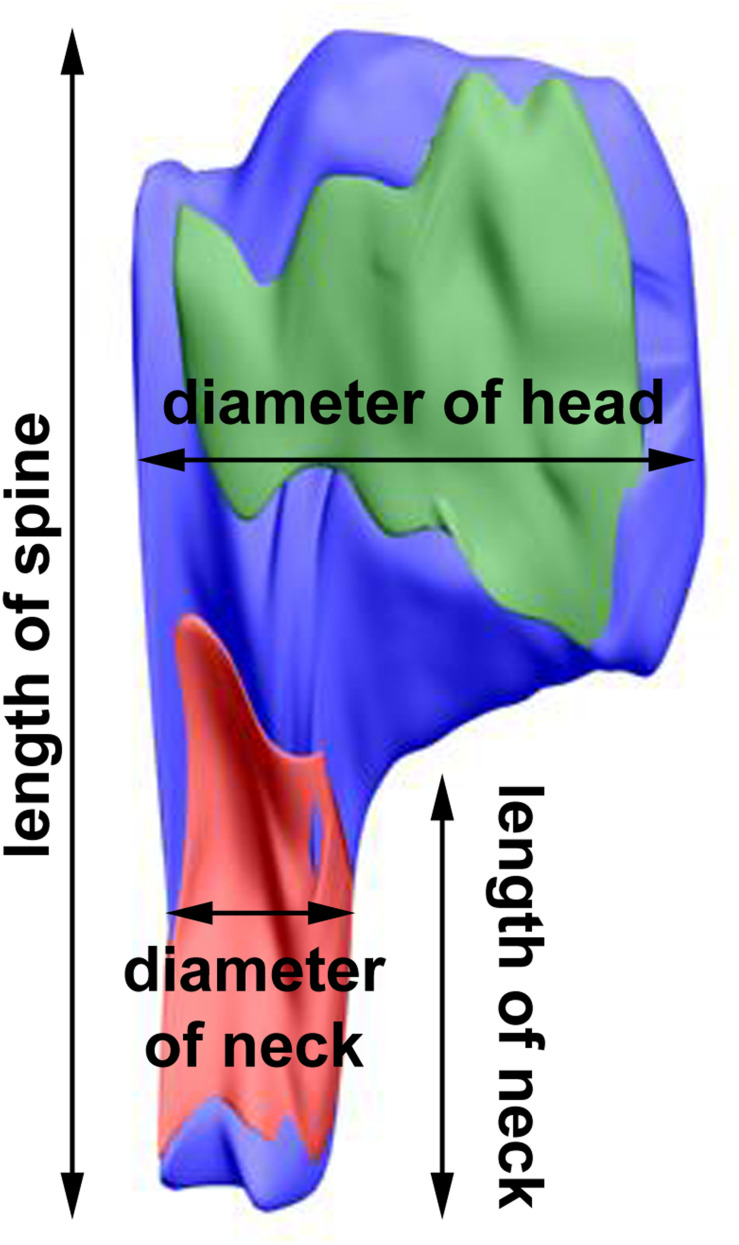
3D reconstruction of a representative double-synapse spine showing the measured parameters. Red area, inhibitory synapse; green area, excitatory synapse.

The length of the spine and of its neck was measured after 3D reconstruction of the spine (Jasinska et al., 2016; Figure 2). In the electron micrographs, total spine area and the areas of spine head and neck were measured, and on the basis of areas in serial sections and according to the known thickness of the sections, volumes of the structures were calculated. The diameter of spine head was measured as the longest diameter parallel to PSD (Bourne and Harris, 2011). Three measurements of neck diameter at different levels were made and averaged (Arellano et al., 2007; Jasinska et al., 2016). The areas of PSD were calculated according to Ostroff et al. (2002). The polyribosomes were counted in each spine, and their number per spine was assessed (Jasinska et al., 2013, 2016). Using 3D reconstructions, distribution of polyribosomes in the spines (location in the head or neck) was estimated.

All measurements were performed using NIH ImageJ software (Analyze-Measure, Cell Counter Plugin; see text footnote 2).

The counting and measurements were done blind—the observer did not know whether the micrographs were taken from LD or DD and ACTIVE or REST groups.

### Statistical Analysis

All data were analyzed using GraphPad Prism 5.01 software (GraphPad Software Inc., United States).

To compare the combined effect of animals’ activity and light conditions on all morphological measurements (total spine volume, the volume of spine head and neck, the length of spine and spine neck, the diameter of spine head and neck, the area of PSD, the number of polyribosomes), 2-way analysis of variance (ANOVA) with *post hoc* Bonferroni test preceded by Kolmogorov–Smirnov normality test was used. Differences in the location of polyribosomes in dendritic spines between REST and ACTIVE groups were also compared with the use of that test. To compare the combined effect of animals’ activity and spine content on the selected parameters (the length of single-synapse spine and its neck, the diameter of double-synapse spine head and neck, PSD area of excitatory and inhibitory synapses of double synapse-spines, the number of polyribosomes in double-synapse spines), 2-way ANOVA with *post hoc* Bonferroni test preceded by Kolmogorov–Smirnov normality test was used. To facilitate the evaluation of statistically significant results, the Cohen effect size (Cohen *d*; Lakens, 2013) was calculated.

To test the relationships between the single- and double-synapse spine parameters that change over the course of a day and under different light conditions, Pearson correlation coefficient was used.

In *Results* and in graphs, data are presented as means ± SD and means ± SEM, respectively.

## Results

### Volume of Dendritic Spines

No significant circadian or diurnal changes were observed in the total volume [single: 2-way ANOVA, *F*_*phase*_(1,197) = 0.66, *P* = 0.417; double: 2-way ANOVA, *F*_*phase*_(1,85) = 2.62, *P* = 0.109], as well as in the volume of spine heads [single: 2-way ANOVA, *F*_*phase*_(1,197) = 0.74, *P* = 0.391; double: 2-way ANOVA, *F*_*phase*_(1,85) = 1.52, *P* = 0.220] and necks [single: 2-way ANOVA, *F*_*phase*_(1,197) = 0.25, *P* = 0.616; double: 2-way ANOVA, *F*_*phase*_(1,85) = 2.01, *P* = 0.160] in single- and double-synapse spines under LD and DD conditions. Similarly, there were no differences in the total volume [single: 2-way ANOVA, *F*_*condition*_(1,197) = 1.36, *P* = 0.245; double: 2-way ANOVA, *F*_*condition*_(1,85) = 0.21, *P* = 0.644], the volume of spines heads [single: 2-way ANOVA, *F*_*condition*_(1,197) = 2.55, *P* = 0.112; double: 2-way ANOVA, *F*_*condition*_(1,85) = 0.05, *P* = 0.827] and necks [single: 2-way ANOVA, *F*_*condition*_(1,197) = 0.003, *P* = 0.956; double: 2-way ANOVA, *F*_*condition*_(1,85) = 0.40, *P* = 0.531] in single- and double-synapse spines between LD and DD conditions.

### Total Length of Dendritic Spines and Length of Spine Necks

**FIGURE 3:**
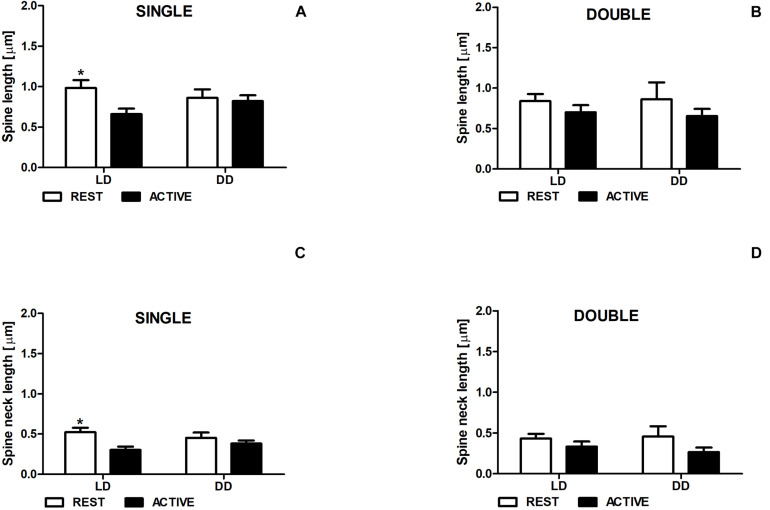
Changes of the length of single-synapse spines and their necks are driven by the light. Spine length **(A,B)** and spine neck length **(C,D)** in REST and ACTIVE groups under LD and DD conditions. All graphs show means ± SEM (2-way ANOVA, *P < 0.05). *N* = 4 mice per group.

In the LD group, single-synapse spines were moderately longer (Cohen *d* = 0.58) in the subgroup REST compared to ACTIVE [2-way ANOVA, *P* < 0.05, *t* = 2.263; *F*_*phase*_(1,197) = 3.87, *P* = 0.051; Figure 3A]. In addition, we found similar differences in the length of single-synapse spine necks between the subgroups REST and ACTIVE [medium effect size, Cohen *d* = 0.68; 2-way ANOVA, *P* < 0.05, *t* = 2.663; *F*_*phase*_(1,197) = 1.89, *P* = 0.171; Figure 3C].

On the contrary, no significant differences in the length of single-synapse spines and spine necks between the subgroups REST and ACTIVE were observed in the DD group (Figures 3A,C).

In both groups (LD and DD), no significant differences between the phases were observed in the length of double-synapse spines or in the length of double-synapse spine necks [REST/ACTIVE; 2-way ANOVA, spine length: *F*_*phase*_(1,85) = 1.90; *P* = 0.172, spine neck length: *F*_*phase*_(1,85) = 3.35, *P* = 0.071; Figures 3B,D].

There were also no differences in the parameters between LD and DD groups in both single-synapse [2-way ANOVA, spine length: *F*_*condition*_(1,197) = 0.05, *P* = 0.827, spine neck length: *F*_*condition*_(1,197) = 0.009, *P* = 0.923] and double-synapse spines [2-way ANOVA, spine length: *F*_*condition*_(1,85) = 0.008, *P* = 0.927; spine neck length: *F*_*condition*_(1,85) = 0.07, *P* = 0.787; Figures 3A–D].

### Diameter of Spine Heads and Necks

**FIGURE 4:**
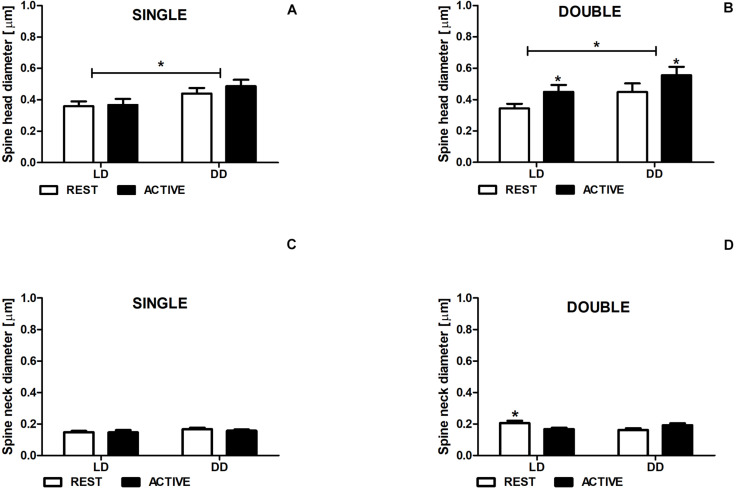
Neck diameter changes in double-synapse spines are driven by the light. Diameter of spine heads **(A,B)** and necks **(C,D)** in groups REST and ACTIVE under LD and DD conditions. All graphs show means ± SEM (2-way ANOVA, *P < 0.05). *N* = 4 mice per group.

In both LD and DD groups, the diameters of single-synapse spine heads did not differ between the subgroups REST and ACTIVE [2-way ANOVA, *F*_*phase*_(1,197) = 0.50, *P* = 0.480; Figure 4A], whereas the diameters of double-synapse spine heads were moderately (Cohen *d* = 0.56) and slightly (Cohen *d* = 0.41) larger in the ACTIVE phase compared to REST in the LD and DD groups, respectively [2-way ANOVA, *F*_*phase*_(1,85) = 4.61, *P* = 0.034; Figure 4B].

Significant differences were found in the head diameters of single- [2-way ANOVA, *F*_*condition*_(1,197) = 6.24, *P* = 0.013] and double-synapse spines between LD and DD conditions [2-way ANOVA, *F*_*condition*_(1,85) = 4.53, *P* = 0.036; Figures 4A,B].

**FIGURE 5:**
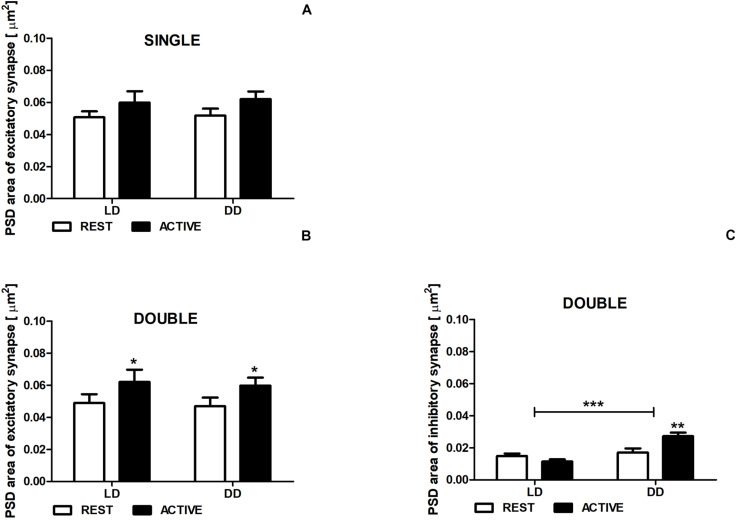
Changes of PSD area of inhibitory synapses in double-synapse spines are driven by both circadian clock/locomotor activity and light. PSD area of excitatory **(A,B)** and inhibitory synapses **(C)** in groups REST and ACTIVE under LD and DD conditions. All graphs show means ± SEM (2-way ANOVA, *P < 0.05, **P < 0.01, ***P < 0.001). *N* = 4 mice per group.

The neck diameter of single-synapse spines did not change between the subgroups REST and ACTIVE irrespectively of the light condition [2-way ANOVA, *F*_*phase*_(1,197) = 2.763, *P* = 0.098; Figure 5C], whereas the neck diameter of double-synapse spines was moderately larger (Cohen *d* = 0.76) in the subgroup REST than in the subgroup ACTIVE only in LD conditions [2-way ANOVA, *P* < 0.05, *t* = 2.336; *F*_*interaction*_(1,85) = 9.04, *P* = 0.0035; Figure 4D].

There were no differences in the diameters of spine necks between LD and DD groups in both single- [2-way ANOVA, *F*_*condition*_(1,197) = 0.33, *P* = 0.568; Figure 4C] and double-synapse spines [2-way ANOVA, *F*_*condition*_(1,85) = 0.76, *P* = 0.387; Figure 4D].

### Area of PSD

There were no differences in the PSD area of excitatory synapses in single-synapse spines between the subgroups REST and ACTIVE in both LD and DD groups [2-way ANOVA, *F*_*phase*_(1,197) = 3.41, *P* = 0.066], as well as between LD and DD conditions [2-way ANOVA, *F*_*condition*_(1,197) = 0.09, *P* = 0.759; Figure 5A].

The PSD areas of excitatory synapses localized on double-synapse spines were slightly (Cohen *d* = 0.40) and moderately (Cohen *d* = 0.51) larger in the ACTIVE phase compared to REST in the LD and DD groups, respectively [2-way ANOVA, *F*_*phase*_(1,85) = 4.33, *P* = 0.040; Figure 5B), whereas the PSD area of inhibitory synapses was substantially larger (Cohen *d* = 0.88) in the subgroup ACTIVE compared to the subgroup REST in the DD group [2-way ANOVA, *P* < 0.01, *t* = 3.557; *F*_*interaction*_(1,85) = 10.88, *P* = 0.0014; Figure 5C].

Moreover, significant differences in the PSD area of inhibitory synapses were found between LD and DD conditions [2-way ANOVA, *F*_*condition*_(1,85) = 19.38, *P* < 0.0001; Figure 5C], whereas in the PSD area of excitatory synapses localized on double-synapse spines did not differ between LD and DD groups [2-way ANOVA, *F*_*condition*_(1,85) = 0.12, *P* = 0.725; Figure 5B].

**FIGURE 6:**
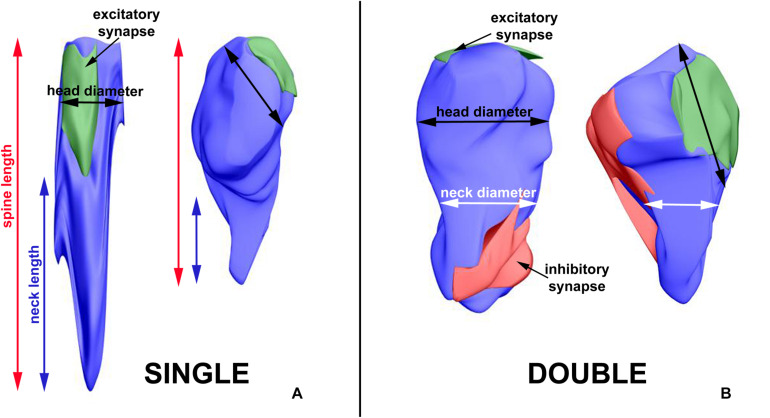
3D reconstructions showing exemplary differences in single-synapse spine length, neck length and head diameter **(A)**, double-synapse spine head and neck diameter and PSD area of excitatory and inhibitory synapses **(B)**. The left sides of A and B—spines from LD REST group, the right sides—from DD ACTIVE group.

The exemplary differences in single-synapse spine length, neck length and head diameter, double-synapse spine head and neck diameter, and PSD area of excitatory and inhibitory synapses are presented in Figure 6.

### Number and Distribution of Polyribosomes in Dendritic Spines

**FIGURE 7:**
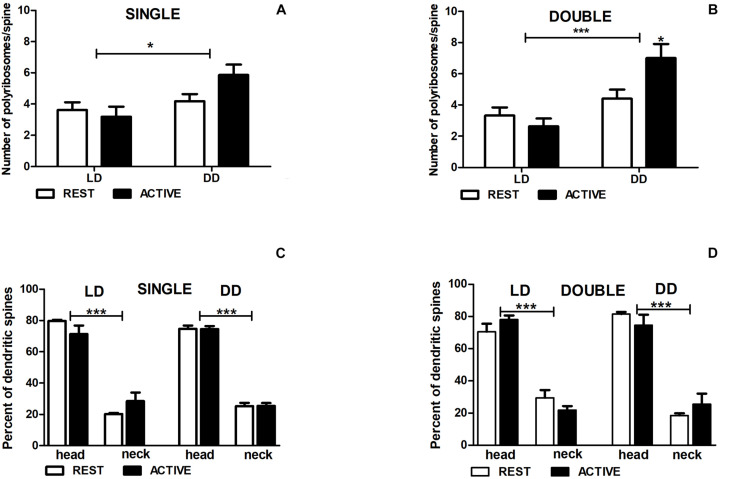
Changes of polyribosome number in double-synapse spines are driven by both circadian clock/locomotor activity and light. Number of polyribosomes in spines **(A,B)** and their distribution **(C,D)** in groups REST and ACTIVE under LD and DD conditions. All graphs show means ± SEM (2-way ANOVA, *P < 0.05, ***P < 0.001). *N* = 4 mice per group.

In the LD group, no significant differences in the number of polyribosomes in single- [2-way ANOVA, *F*_*phase*_(1,197) = 0.97, *P* = 0.326] and double-synapse spines [2-way ANOVA, *F*_*phase*_(1,85) = 1.89, *P* = 0.173] were found between the subgroups REST and ACTIVE (Figures 7A,B).

In the DD group, the number of polyribosomes in double-synapse spines was moderately higher (Cohen *d* = 0.67) in the subgroup ACTIVE than in the subgroup REST [2-way ANOVA, *P* < 0.05, *t* = 2.704; *F*_*interaction*_(1,85) = 5.637, *P* = 0.020; Figure 7B], whereas there were no differences between the subgroups in single-synapse spines [REST/ACTIVE; 2-way ANOVA, *F*_*interaction*_(1,197) = 2.76, *P* = 0.098; Figure 7A].

However, the differences in the number of polyribosomes were found in single- [2-way ANOVA, *F*_*condition*_(1,197) = 6.44, *P* = 0.012] and double-synapse spines [2-way ANOVA, *F*_*condition*_(1,85) = 15.31, *P* = 0.0002] between LD and DD conditions (Figures 7A,B).

Irrespective of spine type and light conditions, the majority of spines contained more polyribosomes in the heads [single: 75.16% ± 2.97%, double: 76.17% ± 4.06%] than in the necks [single—2-way ANOVA, LD REST: *t* = 10.980, *P* < 0.001; LD ACTIVE: *t* = 7.915, *P* < 0.001; *F*_*location*_(1,12) = 178.5, *P* < 0.0001; DD REST: *t* = 17.590, *P* < 0.001; DD ACTIVE: *t* = 17.490, *P* < 0.001; *F*_*location*_(1,12) = 615.0, *P* < 0.0001; double—2-way ANOVA, LD REST: *t* = 7.491, *P* < 0.001; LD ACTIVE: *t* = 10.250, *P* < 0.001; *F*_*location*_(1,12) = 157.4, *P* < 0.0001; DD REST: *t* = 9.358, *P* < 0.001; DD ACTIVE: *t* = 7.292, *P* < 0.001; *F*_*location*_(1,12) = 138.6, *P* < 0.0001]. There were no differences in the distribution of polyribosomes between the subgroups REST and ACTIVE in both light conditions [single—LD: 2-way ANOVA, *F*_*phase*_(1,12) = 0.00, *P* > 0.99; DD: 2-way ANOVA, *F*_*phase*_(1,12) = 0.00, *P* > 0.99; double—LD: 2-way ANOVA, *F*_*phase*_(1,12) = 0.00, *P* > 0.99; DD: 2-way ANOVA, *F*_*phase*_(1,12) = 0.00, *P* > 0.99; Figures 7C,D].

### Correlations of the Number of Polyribosomes and PSD Area of Synapses With Other Spine Parameters

**FIGURE 8:**
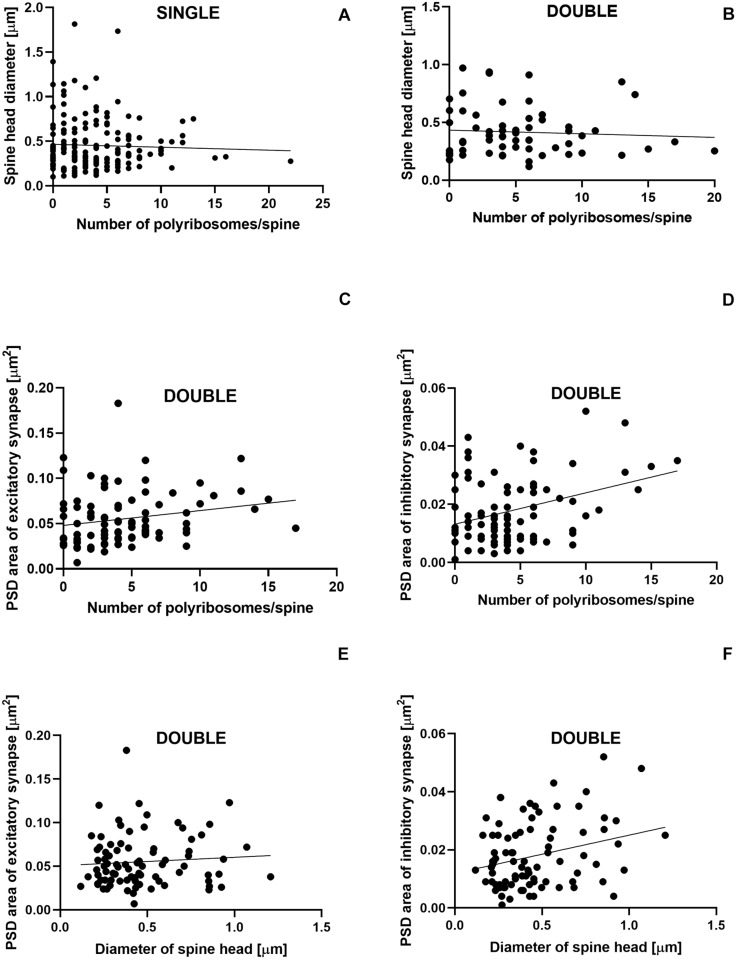
Size of inhibitory synapses located on double-synapse spines is positively correlated with the number of polyribosomes and diameter of spine heads. Correlations between the number of polyribosomes and spine head diameter of single-synapse spines **(A)** and double-synapse spines **(B)** and PSD area of excitatory **(C)** and inhibitory **(D)** synapses on double-synapse spines and between spine head diameter of double-synapse spines and PSD area of excitatory **(E)** and inhibitory synapses **(F)**. *N* = 4 mice per group.

The number of polyribosomes was not correlated with the diameter of spine head in single- (Pearson correlation coefficient, *r* = −0.04, *P* = 0.600; Figure 8A) and double-synapse spines (Pearson correlation coefficient, *r* = −0.066, *P* = 0.605; Figure 8B). Similarly, no correlation was found between the diameter of double-synapse spine head and PSD area of excitatory synapses (Pearson correlation coefficient, *r* = 0.078, *P* = 0.476; Figure 8E); however, such (positive) correlation occurred in case of inhibitory synapses (Pearson correlation coefficient, *r* = 0.277, *P* = 0.009; Figure 8F). Moreover, the number of polyribosomes in double-synapse spines was positively correlated with PSD area of inhibitory synapses (Pearson correlation coefficient, *r* = 0.346, *P* = 0.0009; Figure 8D), but not of excitatory synapses (Pearson correlation coefficient, *r* = 0.204, *P* = 0.056; Figure 8C).

### Relationship Between Circadian Changes in Geometric Parameters and Spine Content

To answer the question whether the content of the spines influences the cyclic changes of their geometric parameters, we selected for further analysis only the parameters that differed between the activity phases of the animals. Single- and double-synapse spines were divided into three subgroups: spines without any organelles (sER-free spines), spines containing sER, and spines containing SA (Jasinska et al., 2019).

**FIGURE 9:**
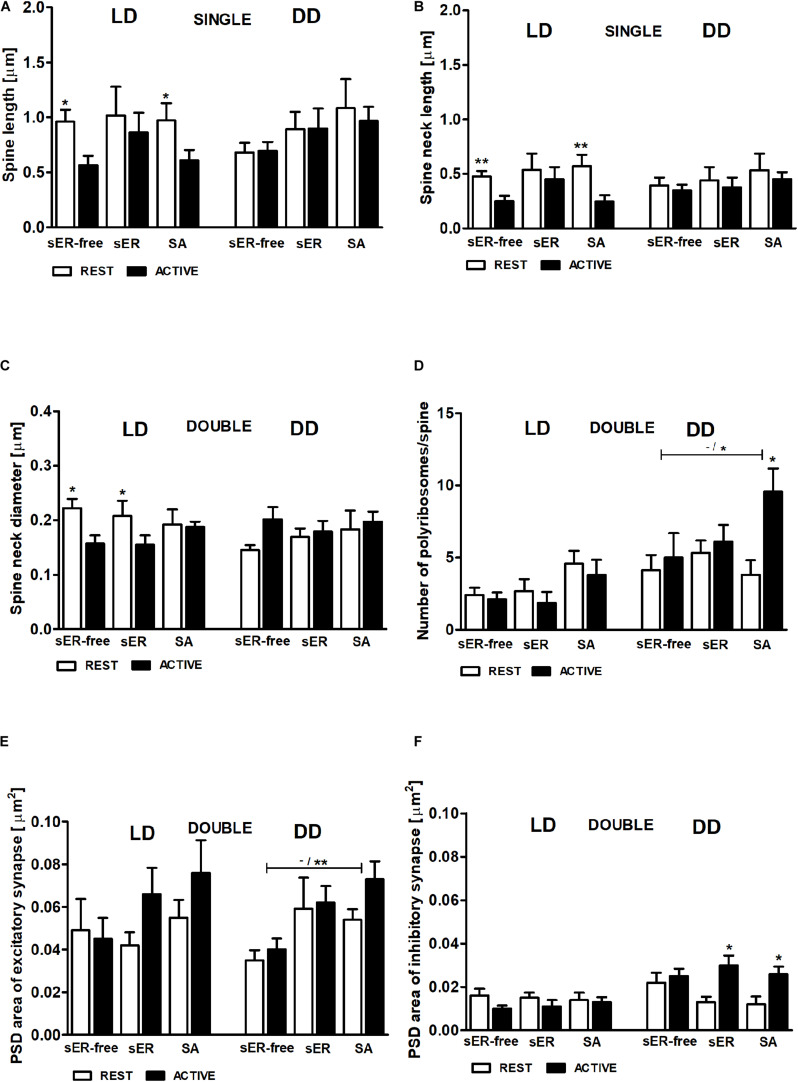
Relationship between geometric parameters and spine content (spines sER-free, containing sER, and containing SA) during diurnal and circadian cycles. Spine length **(A)**, spine neck length **(B)** of single-synapse spines, spine neck diameter **(C)**, number of polyribosomes per spine **(D)**, and PSD area of excitatory **(E)** and inhibitory **(F)** synapses of double-synapse spines in groups REST and ACTIVE under LD and DD conditions. All graphs show means ± SEM (2-way ANOVA, *P < 0.05, **P < 0.01). *N* = 4 mice per group. The asterisks above the bars indicate significant differences in the percentage of spines with the same content between activity phases (REST vs. ACTIVE), whereas the asterisks above lines show significant differences between spines with different contents for the corresponding REST/ACTIVE phase.

In the LD group, total spine length was substantially longer in sER-free single-synapse spines (Cohen *d* = 0.99) and in single-synapse spines containing SA (Cohen *d* = 0.81) in the REST phase compared to ACTIVE [2-way ANOVA, *F*_*phase*_(1,73) = 5.69, *P* = 0.020; Figure 9A]. Similarly, the neck length of single-synapse spines was substantially longer in the spines with the same content (sER-free: Cohen *d* = 1.13, SA: Cohen *d* = 1.12) in the subgroup REST compared to ACTIVE [2-way ANOVA, *F*_*phase*_(1,73) = 8.16, *P* = 0.006; Figure 9B]. Significant differences were also found in the neck diameters of sER-free double-synapse spines (large effect size, Cohen *d* = 1.51) and double-synapse spines containing sER (large effect size, Cohen *d* = 0.94) between the REST and ACTIVE subgroups [2-way ANOVA, *F*_*phase*_(1,37) = 6.51, *P* = 0.015; Figure 9C]. On the other hand, no significant differences in the head diameter of double-synapse spines irrespective of spine content and light conditions were found between the subgroups REST and ACTIVE [2-way ANOVA, LD: *F*_*phase*_(1,37) = 3.01, *P* = 0.091; *F*_*content*_(2,37) = 0.12, *P* = 0.889; DD: *F*_*phase*_(1,41) = 1.18, *P* = 0.285; *F*_*content*_(2,41) = 2.86, *P* = 0.068].

PSD areas of excitatory and inhibitory synapses did not differ irrespective of double-synapse spine content between activity phases in the LD group [2-way ANOVA, *F*_*phase*_(1,37) = 1.76, *P* = 0.192; *F*_*content*_(2,37) = 1.09, *P* = 0.344; Figures 9E,F], whereas in the DD group, double-synapse spines containing SA had substantially larger (Cohen *d* = 1.57) PSD area of excitatory synapses than sER-free double-synapse spines only in the subgroup ACTIVE [2-way ANOVA, *t* = 2.862, *P* < 0.05; *F*_*content*_(2,41) = 5.64, *P* = 0.007; Figure 9E]. Additionally, double-synapse spines containing sER as well as containing SA had substantially larger PSD area of inhibitory synapses during ACTIVE phase compared to REST in the DD group [sER: Cohen *d* = 1.34, *t* = 2.882, *P* < 0.05; SA: Cohen *d* = 1.49 *t* = 2.866, *P* < 0.05; 2-way ANOVA, *F*_*phase*_(1,41) = 10.4, *P* = 0.003; *F*_*content*_(2,37) = 0.12, *P* = 0.889; Figure 9F].

In the LD group, no significant differences in the number of polyribosomes in double-synapse spines with different content were found between the subgroups REST and ACTIVE [2-way ANOVA, *F*_*phase*_(1,37) = 0.77, *P* = 0.387; *F*_*content*_(2,37) = 3.15, *P* = 0.055], whereas in the DD group, the number of polyribosomes in double-synapse spines containing SA was moderately higher (Cohen *d* = 0.67) in the subgroup ACTIVE compared to REST [2-way ANOVA, *t* = 2.758, *P* < 0.05; *F*_*phase*_(1,41) = 4.77, *P* = 0.035], as well as compared with sER-free double-synapse spines in the subgroup ACTIVE [large effect size, Cohen *d* = 0.98; 2-way ANOVA, *t* = 2.417, *P* < 0.05; *F*_*interaction*_(2,41) = 4.63, *P* = 0.011; Figure 9D].

## Discussion

### Single-Synapse Spines

It is known that the changes in synapse strength are reflected by various modifications of spine geometry affecting both, head and neck of the spine (Fifkova, 1985; Araya et al., 2014; Bosch et al., 2014; Nishiyama and Yasuda, 2015). Single-synapse spines showed no change in volume regardless of the phase of the day and light conditions (day/night under LD condition and subjective day/subjective night under DD condition). It was also consistent with the absence of circadian changes in PSD area of excitatory synapses.

On the other hand, the total length and length of the neck of single-synapse spines are driven by light. The length of spine neck can affect the strength of excitatory synapses located on spine heads (Araya et al., 2014). There are no correlations between head size, PSD size, and neck length (Benavides-Piccione et al., 2002; Arellano et al., 2007), indicating that these parameters are independently regulated, and their contributions to synapse strength are not interrelated.

The observed changes in the length of single-synapse spines and the length of their necks concern single-synapse spines without organelles and containing SA. Our previous study showed that single-synapse spines containing SA, regardless of their shape, were not affected by the light (Jasinska et al., 2019). The present results indicate that although light does not change the number of these spines, it could modify their morphology.

In the somatosensory cortex, an increase in the number of excitatory synapses on the spines was found only as the effect of light under LD conditions, whereas an increase in the number of single-synapse spines was observed both, in the light phase of LD regime and during the subjective day under DD conditions (Jasinska et al., 2014; Jasinska et al., 2015). This study shows that the light also promotes elongation of single-synapse spine necks. Interestingly, under constant darkness the number of excitatory synapses on all spines does not change (Jasinska et al., 2015), and the necks of single-synapse spines do not elongate.

However, during the subjective day, there are more single-synapse spines, what probably results from double- to single-synapse spines transformation and requires the removal of inhibitory synapses from double-synapse spines (Jasinska et al., 2015). The number of single-synapse spines is controlled by the biological clock (Jasinska et al., 2015), whereas the efficiency of excitatory synapses on the spines seems to be regulated differently, depending on the light conditions: under LD conditions by changes in spine geometry and in constant darkness by cyclic formation and degradation of inhibitory synapses as well as transformation between single- and double-synapse spines.

It could be supposed that the changes in spine geometry are much faster than the process of formation and breakdown of inhibitory synapses; hence, the stress, such as the light for nocturnal animals, supports spine modification allowing more rapid execution of excitatory signaling.

Moreover, there were differences in the diameters of the single-synapse spine heads, which were larger in constant darkness compared to LD, although this effect was not accompanied by an increase in the head volume (suggesting the change of head shape) or in PSD area of excitatory synapses. Similar differences between the light conditions were observed in the number of polyribosomes in single-synapse spines, but these parameters were not correlated.

### Double-Synapse Spines

In double-synapse spines, no changes were observed in the total volume of spines or in the volume of the spine heads and necks regardless of light condition. On the other hand, the diameter of the neck increased during the day. The studies related to activity-dependent neural plasticity have shown that the increase in the diameter of spine neck is usually accompanied by its shortening (Fifkova and Anderson, 1981; Jasinska et al., 2006; Tønnesen et al., 2014). These morphological parameters of the neck are not correlated with each other (Arellano et al., 2007), but both reduce a resistance of the neck. Accordingly, in our study, although an increase in the neck diameter was observed, no changes were found in the length of the neck of double-synapse spines during the day irrespective of the light conditions. The day/night (under LD conditions) changes in the neck diameter were observed in double-synapse spines without sER/SA and containing sER, but not in double-synapse spines containing SA. This is in line with the fact that spines containing SA are considered to be the most stable spines (Knott et al., 2006; Bourne and Harris, 2008).

Although the modifications of the size of single-synapse spine neck could directly change (reduce or increase) the signal of the excitatory synapse, double-synapse spines also contain on the neck the inhibitory synapse, which might additionally regulate excitatory synaptic transmission. During the day, the decrease in the number of double-synapse spines (Jasinska et al., 2015) and a simultaneous increase in the size of their neck areas could be considered as a mechanism balancing the fewer number of spines by enhancing the efficiency of their synapses.

Another interesting result is an increase in the diameter of spine heads in DD compared to LD conditions, observed in both types of spines; additionally, in double-synapse spines, there were differences between the day/subjective day and night/subjective night irrespective of spine content. PSD enlargement is usually correlated with enlargement of the spine head (Knott et al., 2006; Arellano et al., 2007), and it seems that changes of the diameter of double-synapse spine heads should be related to the modifications of excitatory synapses, because they are located on the heads, and their PSD area is modified, depending on the activity phase of the animals. Unexpectedly, these parameters are not correlated, whereas a positive correlation was found between the head diameter and the PSD area of inhibitory synapses. This result is especially interesting, because inhibitory synapses are usually located on the necks of double-synapse spines (Jasinska et al., 2006). Moreover, enlargement of PSD area in inhibitory synapses was accompanied by increase in the number of polyribosomes, suggesting an increase in the strength of these synapses observed only during the subjective night. The size of inhibitory synapses changed only in double-synapse spines containing sER or SA. Moreover, in double-synapse spines containing SA, synapse enlargement was accompanied by increase in the number of polyribosomes. The SA, in cooperation with polyribosomes, is responsible for the enlargement of spine head and increased accumulation of glutamatergic receptors, leading to enhancement of excitatory synapses (Vlachos et al., 2009; Ostroff et al., 2010; Jedlicka and Deller, 2017). It seems that SA could play a similar role in double-synapse spines and their inhibitory synapses during the circadian cycle. Similarly, the presence of sER in spines contributes to synaptic enlargement (Chirillo et al., 2019).

Table 1 summarizes the influence of the light and endogenous effect on the number and morphology of dendritic spines in mouse barrel cortex.

**TABLE 1:** Influence of light and the circadian clock/locomotor activity on single- and double-synapse spines in mouse barrel cortex.

	**Single-synapse spines**	**Double-synapse spines**
Effect of light	Increase in: number of intermediate sER-free spines (Jasinska et al., 2019), total length of spine (especially sER-free and containing SA), length of spine neck (especially sER-free and containing SA)	Increase in: stubby spines containing sER, mushroom spines containing SA, thin/mushroom spines (Jasinska et al., 2019), diameter of spine neck (especially sER-free and containing sER)
	Decrease in: number of stubby sER-free spines (Jasinska et al., 2019), diameter of spine head, number of polyribosomes	Decrease in: number of stubby sER-free spines, number of mushroom spines (especially sER-free) (Jasinska et al., 2019), diameter of spine head, PSD area of inhibitory synapse, number of polyribosomes
Effect of circadian clock/locomotor activity (endogenous effect)	Increase in: number of thin spines containing sER (Jasinska et al., 2019)	Increase in: total number of spines (Jasinska et al., 2015), number of mushroom spines containing sER (Jasinska et al., 2019), diameter of spine head, PSD area of excitatory synapse, PSD area of inhibitory synapse (especially containing sER and SA) number of polyribosomes (especially in spines containing SA)
	Decrease in: total number of spines (Jasinska et al., 2015), number of stubby spines containing SA (Jasinska et al., 2019)	Decrease in: number of thin spines containing sER, number of mushroom sER-free spines (Jasinska et al., 2019)
Effect of both factors (light and endogenous effect)	Increase in: number of stubby spines containing sER, number of mushroom spines (especially containing sER; Jasinska et al., 2019)	Increase in: number of thin spines (especially sER-free; Jasinska et al., 2019)

### Functional Significance of Changes in Spine Geometry

Results of this study show that single- and double-synapse spines are differently involved in diurnal and circadian changes. Both types of spines regulate synaptic transmission by modifications of the morphology of their necks; however, different geometrical parameters are changed. In single-synapse spines, the neck length modification was observed, whereas double-synapse spines changed the diameter of their necks. Both types of changes are associated with the light phase of the diurnal cycle and have similar effects: regulate the resistance of spine neck, influence the degree of isolation of the spine from its parent dendrite, and even control the effectiveness of excitatory synapse placed on the spine head (Arellano et al., 2007; Araya et al., 2014). However, the changes observed in single- and double-synapse spines have opposite results: elongation of the neck in single-synapse spine increases the isolation of spine head from dendrite, whereas widening of the neck in double-synapse spine increases communication between spine and dendrite. On the other hand, spines with wider and shorter necks might be less susceptible to subsequent changes, because second messengers diffuse faster into the dendritic shafts due to increased capacity of the spine neck, whereas additional depolarization is stronger in spines better isolated from dendrites (with longer and more narrow necks; Tønnesen et al., 2014; Tønnesen and Nägerl, 2016).

Although the length and diameter of spine neck are not correlated with each other (Arellano et al., 2007), in case of experience-dependent changes these parameters have been observed as mutually coupled; i.e., widening of spine neck was accompanied by its shortening (Jasinska et al., 2006; Tønnesen et al., 2014). Such effect was not observed in this study. It could be concluded that modification of only one of neck parameters does not disturb function of neurons of the somatosensory cortex in the diurnal cycle.

The number of excitatory synapses located on single-synapse spines increases during the subjective day (Jasinska et al., 2015), but they do not change their size, while during the subjective night, the number of inhibitory synapses increases (Jasinska et al., 2015), and they are larger. Generally, no morphological changes of the excitatory synapses located on single-synapses spines were observed in the diurnal and circadian cycle. On the contrary, PSD area of inhibitory synapses increased during the subjective night, indicating enhancement of the strength of these synapses. Such enhancement might also have more complex consequence, because strong inhibitory synapses located on double-synapse spines could more efficiently control/regulate the effectiveness of excitatory synapses, affecting the conductivity of the whole spine.

The previous study showed that the number of mushroom double-synapse spines containing SA, i.e., the most mature and stable spines, increases in the light (Jasinska et al., 2019). Interestingly, in constant darkness, the increase in the size of inhibitory synapses correlated with the increase in the number of polyribosomes during the subjective night also concerns double-synapse spines containing SA. This suggests that double-synapse spines with SA are regulated at various levels by both light and endogenous factor and play a special role in diurnal and circadian rhythms.

As appears from this and previous studies (Jasinska et al., 2015, 2019), the function of single- and double-synapse spines in the somatosensory cortex is different during diurnal and during circadian cycle. It seems that in the circadian cycle (under DD conditions), regulation of synaptic transmission is largely based on the inhibitory synapses. Even the changes in the number of single-synapse spines seem to be the consequence of addition/subtraction of inhibitory synapses on double-synapse spines. In the diurnal cycle (under LD conditions), however, both single- and double-synapse spines are equally involved in the regulation of the received inputs, although they regulate them in different ways.

### Implications for Sleep Theories

The synaptic homeostasis hypothesis (Tononi and Cirelli, 2006) presumes strengthening of the synaptic network during the activity phase of animals and weakening during the rest phase. It seems that an opposite trend occurs in the barrel cortex. By enhancing inhibitory transmission in the night/subjective night, overall synaptic transmission is weakened during higher activity of animals.

The results obtained in this study seem to confirm the homeostatic synaptic plasticity hypothesis (Turrigiano and Nelson, 2000; Pozo and Goda, 2010), which assumes the existence of an adaptive compensatory mechanism. In the night/subjective night, the number of inhibitory synapses on the spines increases (Jasinska et al., 2015), and although only during constant darkness, the inhibitory synapses are larger, what is associated with an increase in their strength and consequently leads to weakening of synaptic transmission, being a compensation for excitation resulting from increased activity of animals.

The elongation of single-synapse spines and their necks observed during the day (light phase) under LD conditions also leads to reduced excitatory transmission from spine to the parent dendrite. Interestingly, the number of excitatory synapses increases in the light phase of the diurnal cycle (Jasinska et al., 2015), but due to changes in synapse morphology they are less effective. It seems that the presence of more excitatory synapses during that phase suggests preparation for more stressful conditions but not necessarily does mean a direct increase in excitation. Additionally, the widening of the necks in double-synapse spines has similar significance, because it leads to easier control of excitatory transmission by the inhibitory synapses also present on these spines.

### Study Strengths, Limitations, and Future Research Directions

This study shows for the first time the circadian changes of measurable spine geometric parameters using a rigorous quantitative approach offered by transmission electron microscopy. However, it has certain limitations. We have not performed an *a priori* power analysis, and although the number of spines in the groups is large, we cannot be sure whether it is sufficient to show all existing differences between the groups. Nevertheless, we selected to the study all spines that were completely contained within the sample volume and could serve for 3D reconstruction.

Another limitation of our study is the selection of just two time points across the 24-h cycle. Although they correspond well to changes in animal activity (REST/ACTIVE), we cannot separate the role of the biological clock from the direct influence of the locomotor activity of animals. Further studies using additional time points or another approach (e.g., clock mutants) are necessary to elucidate that question.

This study was conducted on dendritic spines obtained exclusively from the barrel field of the somatosensory cortex. Because significant differences in the cyclic changes of dendritic spine density were found depending on regions of the brain and even between the hemispheres, our results cannot be generalized to the entire neocortex. It seems appropriate to investigate dendritic spines in other regions of the cortex, e.g., in the visual cortex, where light plays a different role. Such comparison of cyclic changes in the geometric parameters of spines between different areas of the brain would significantly increase our understanding of the diurnal and circadian rhythms.

## Data Availability Statement

All datasets presented in this study are included in the article.

## Ethics Statement

The animal study was reviewed and approved by Animal Care and Use Committees of the Jagiellonian University.

## Author Contributions

MJ and EP contributed to conception and design of the study. MJ and OW performed the experiments. MJ and EJ-G analyzed the data. MJ performed the statistical analysis. JL and GL participated in data interpretation. MJ wrote the draft of the manuscript. EP and JL revised the manuscript critically for important intellectual content. All authors contributed to the article and approved the submitted version.

## Conflict of Interest

The authors declare that the research was conducted in the absence of any commercial or financial relationships that could be construed as a potential conflict of interest.

## Footnotes

1 realterm.sf.net

2 http://rsb.info.nih.gov/ij/
